# Smart Multi-Sensor Platform for Analytics and Social Decision Support in Agriculture

**DOI:** 10.3390/s20154127

**Published:** 2020-07-24

**Authors:** Titus Balan, Catalin Dumitru, Gabriela Dudnik, Enrico Alessi, Suzanne Lesecq, Marc Correvon, Fabio Passaniti, Antonella Licciardello

**Affiliations:** 1 Atos Convergence Creators, 500090 Brasov, Romania; catalin.dumitru@atos.net; 2 Centre Suisse d’Electronique et de Microtechnique, 2000 Neuchâtel, Switzerland; gabriela.dudnik@csem.ch (G.D.); marc.correvon@csem.ch (M.C.); 3 ST Microelectronics, Ct-95129 Catania, Italy; enrico.alessi@st.com (E.A.); fabio.passaniti@st.com (F.P.); anto.licciardello@st.com (A.L.); 4 University Grenoble Alpes, CEA, LIST, F-38000 Grenoble, France; suzanne.lesecq@cea.fr

**Keywords:** sensor, data analytics, social feedback, gas sensor, data logger, agriculture, decision support system, Social IoT

## Abstract

Smart agriculture based on new types of sensors, data analytics and automation, is an important enabler for optimizing yields and maximizing efficiency to feed the world’s growing population while limiting environmental pollution. The aim of this paper is to describe a multi-sensor Internet of Things (IoT) system for agriculture consisting of a soil probe, an air probe and a smart data logger. The implementation details will focus of the integration element and the innovative Artificial Intelligence based gas identification sensor. Furthermore, the paper focuses on the analytics and decision support system implementation that provides farming recommendations and is enhanced with a feedback loop from farmers and a social trust index that will increase the reliability of the system.

## 1. Introduction

Increasing demand for food production to feed the ever-growing population requires the augmentation of crop yield to produce more raw materials on a surface of arable land and permanent crops, worldwide [1]. Agriculture intensification (mainly via pesticides and chemical fertilizers) degrades the soil from physical, chemical, and biological point-of-views, leading to soil fertility decline. Moreover, intensive agriculture (i.e., deep soil cultivation, mineral fertilizers, pesticides, and little organic matter supply) is associated with a decrease in soil biodiversity [2].

Monitoring soil nutrients (typically N, P, K) helps to adapt the soil fertilization procedure to the real plant demand, in real time [3]. Several soil sensor systems have been already developed [4], yet none has been widely adopted for fertilizer management due to various technology-specific limitations.

Agricultural activity represents a major source of greenhouse gas (GHG) emissions due to the application of nitrogen-based fertilizers and animal manures, thus contributing to climate change.

**Figure 1:**
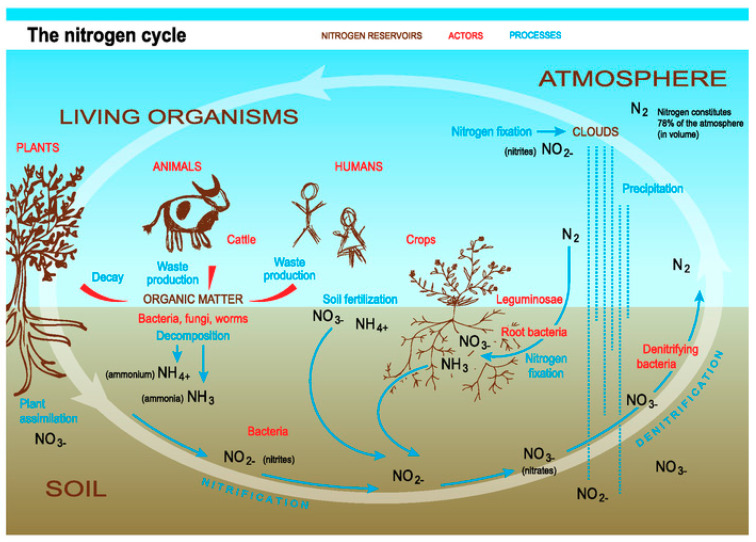
Global nitrogen cycle [5].

For nitrous oxide, quantification of emissions is constrained by the absence of low cost, high frequency measurement systems that can be used within a field environment. Manure decomposition also produces methane. Moreover, ammonia has an adverse impact on human health. Figure 1 provides an overview of the nitrogen cycle in agriculture.

Monitoring soil quality with focus on yield should be doubled by gaseous emissions monitoring in case of sustainable agriculture.

Furthermore, fertilizers efficiency at crop level, as well as gaseous emissions levels are dependent on different parameters like weather, soil type, seed type, moisture, compactness, etc., that need to be taken into consideration by a decision support system, as they are too diverse to be assessed without additional processing.

Agricultural decision support systems (AgriDSS) help farmers and their advisors in their decision-making, in particular regarding plant nutrition and treatment of pests [6]. These decision support systems could use [7]:

- The usage of existing or new sensors that provide information of in situ soil nutrients and adversarial gaseous emissions from fertilizer decomposition;
- New models for data analytics and recommendation decisions to support the farmers activity;
- Efficient IoT communication and integration;
- Reliable prediction models and social feedback.

The term social feedback mentioned above refers to interaction with the users, the farmers in this case, that validate or give feedback to the decision support system, thus participating in the learning and refining process of the algorithm. Furthermore, feedback is used for evaluating the reliability of the data sources, thus minimizing the impact of erroneous elements. 

The paper presents a multi-sensor IoT prototype node that will measure in-field the soil nutrients and gaseous emissions just above the soil. There are three main generic parts of the multi-sensor platform: the sensing, the integration and communication part and the data analytics part. From the overall project realization, though we will present the generic hardware and software architecture, we have chosen to focus in this paper on some elements that are illustrative for the overall system functionality:

- From the sensing part we have chosen to detail the so-called air probe that measures gaseous emissions that includes an innovative AI based gas recognition method;
- From the integration part, the node called Smart Data Logger (SDL) and on the communication path from this integrative node to the Back End Server (BES) are detailed;
- The analytics and decision support system that provide advices to the farmer about the fertilization strategy is also presented. The decision support model provides advice to farmers based on the conceptual plant model (the stage for the plant growth), the multi-sensor values and meteorological prognosis, taking into consideration also possible environmental aspects. Examples of recommendation cover classical farming actions, e.g., to use a certain type of fertilizer or the need for irrigation.

This paper is organized as follows: Section 2 summarizes the related work; Section 3 describes the generic architecture of the multi-sensor secure connected node, as well as physical, mechanical, power and communication constraints that influenced the design of the system. Following the overall system description, the paper focuses on the air probe. The IoT communication path is also described; Section 4 is dedicated to the analytics and decision support system. Note that feedback from the farmer is of great interest to improve the overall system performance. System integration and preliminary results are given in Section 5 while Section 6 summarizes the main outcomes and future work directions.

## 2. Sensor Based eAgriculture

Food production must increase worldwide to feed the ever-increasing world population. In parallel, there is increasing concern about chemical plant nutrient losses to the environment, leading to water and air pollution [8].

While losses of nutrients through the ground are classically mentioned, emissions to the atmosphere are also a source of loss. Exceeding nitrogen provided by fertilizers can be converted to nitrous oxide (N_2_O) through the nitrification-denitrification process [9]. Nitrous oxide is a very potent greenhouse gas, with 310 times greater global warming potential than carbon dioxide (CO_2_). Nitrous oxide can be produced in soils following fertilizer application, both chemical and organic. Production of methane (CH_4_) comes from manure decomposition. This gas possesses a global warming potential 21 times higher than CO_2_ [10]. Lastly, ammonia (NH_3_) can be also produced, with an adverse impact on human health [11]. Therefore, monitoring these gaseous emissions just above the ground, directly in the field, will help the farmer properly manage fertilizer application, without losses to the environment.

Metal Oxide Semiconductor (MOS) gas sensors are suitable for many applications in different market segments. They are considered low cost in comparison with other sensing technologies. They are well-known for the high sensitivity and poor selectivity [12]. The air probe developed in the course of the project is based on STMicroelectronics gas sensor. It also integrates other sensors like relative humidity and temperature to the volatile organic compound (VOC) sensing channel in a unique combo device together with an application specific integrated circuit (ASIC). The potential of power reduction, low noise, enhancement of selectivity by detecting the VOC fingerprints constitute some advantages that this integration is offering to the air probe functionalities [13,14,15]. Specifically, the air probe is designed to detect the gas concentration of target gasses such as ammonia, methane and nitrogen dioxide by the temperature modulation approach [16,17] and the application of artificial neural network multi-layer perceptron. This control and process chain can potentially transform a low cost sensor into a powerful tool for sensor based eAgriculture.

Several commercial decision support systems already exist on the market. The John Deere Field Connect implementation [18] is monitoring only soil moisture, temperature, humidity, wind speed and direction. However, it is not monitoring soil nutrients. Method Smart Fertilizer Software [19] describes crop monitoring based on pre-existing soil testing results but it is not performing in situ nutrient status monitoring. Implementation Farmer Pro [20] offers a fertilizer control system and prescription maps to increase yield. Other systems are based on satellite images for crop monitoring, but they do not actually monitor the soil conditions.

The objective of the project, called SARMENTI, is to develop a multi-sensor, low power IoT secure node to provide decision support to farmers by monitoring in real-time and in situ soil nutrients and gaseous emission just above the ground over the whole crop lifecycle. More precisely, the SARMENTI node will measure soil macronutrients N, P and K in their ionic form of NO_3_, NH_4_, PO_4_ and K thanks to a “soil probe” buried in the soil. The soil probe will integrate electrochemical sensors, namely, nano-wire sensors together with potentiometric ISE sensors, taking advantage of measurement redundancy and specific property of each technology. A hygroscopic membrane will attract water from the soil, avoiding integration of a power-hungry active pump usually used to exact water from a soil sample. The SARMENTI node will also measure methane and ammonia emissions with Metal Oxide Semiconductor (MOS) gas sensors coupled with edge-AI techniques for gas recognition. From the sensing part, this paper will focus on presenting the details of the gaseous sensors.

Sensors will be configured to measure on a daily basis over crop lifecycles and sent data to the cloud for further analysis, based on the dedicated platform that will be presented in Section 4. The application that we have developed, called “SARMENTI Farm Advisor”, optimized also for mobile devices, focuses on providing real-time predictions and prescriptions to the farmer. Then, the farmer will timely perform appropriate actions regarding fertilization, with direct impact on crop growth, soil quality and water usage and farmer income.

## 3. Development of a Smart-Multi-Sensor Secure Connected Node

**Figure 2:**
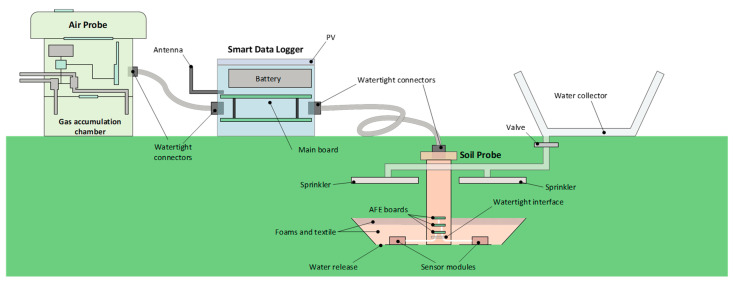
Secure Connected Node physical design.

The SARMENTI secure connected node is split into three main parts (see Figure 2):

- A Soil Probe buried in the soil [21], containing electrochemical sensors (nanowire and ISE) in a hygroscopic membrane to monitor soil nutrient concentrations in real-time;
- An Air Probe that collects information regarding environmental conditions and gaseous emissions (esp. [NH_3_], [N_2_O], [CH_4_]) just above the ground [22];
- A secure Smart Data Logger connected to the IoT [23]. It will collect data from the Air and Soil Probes and send them to the cloud.

Cyber-security is also addressed in the SARMENTI work program, hence addressing the “Insecure design and/or development” gap, and “Lack of interoperability across different IoT devices, platforms and frameworks” gap highlighted in the Baseline Security Recommendations for IoT report. Finally, SARMENTI “closes the loop” with decision advice and advanced analytics located in the cloud.

The Air Probe and the Soil Probe are linked to the Smart Data Logger by means of a watertight cable that transmits the power supply and the communication lines.

The Air Probe and the Smart Data Logger are located on the soil surface, with a fixation to ensure the devices stay in place. The Soil Probe is buried in the soil at an adaptable depth that depends on the type of crop and soil. The need of addition of a rain water collector, able to transmit the water close to the Soil Probe, is optional and is still being analyzed.

The power supply provided by a rechargeable battery combined with a photovoltaic (PV) cell, a harvester and a battery charger, are part of the Smart Data Logger. In this regard, the Smart Data Logger is in charge of: (i) implementing the harvester; (ii) charging the battery and; (iii) monitoring the battery capacity. The Smart Data Logger generates the different supplies required by its own circuits, and provides independent supplies to the Air Probe and to the Soil Probe, taking care also of the power saving policy.

The Smart Data Logger collects the measurements that both the Air Probe and the Soil Probe generate, either on demand or in a scheduled way. It also stores the received data together with a timestamp and sensor status information. The data is sent on demand to the farmer Mobile Device, when the farmer goes close to the node and requests it for information, or sent in a scheduled daily basis to the Back-End Server by means of the LoRa infrastructure, depending on the use case.

The Air Probe measures environmental variables and gas emissions in the air close to the soil surface. It is powered by the Smart Data Logger, sending the measured values to it upon request.

The Soil Probe measures the nutrient variables in the soil at a depth that depends on the type of crop and soil. It is also powered by the Smart Data Logger, sending the measured values to it upon request.

Each module and the link in between is watertight, with enough ingress protection to be able to work in harsh conditions: (i) dust; (ii) humidity; (iii) rain and; (iv) on or in soils totally or partially flooded for long periods of time.

We will further shortly introduce the Soil Probe. Then, the Air Probe and the Smart Data Logger will be described in details.

### 3.1. Soil Probe

The Soil Probe has several constraints to consider regarding the sensors, the electronic front-ends and the water management:

- Given that the sensors are linked by a conductive media, there might be leakage current between them, independently of how well and isolated the front-ends are conceived;
- Each sensor requires individual calibration because the manufacturing cannot ensure a repetitive production;
- Such calibration does not last all along the sensor lifetime, so there is a need for periodic recalibration;
- In order for an electrochemical sensor to measure properly, it requires the surface be wetted by the soil humidity bringing the nutrients to be measured;
- After the measurement is done, the water or humidity on the sensor surface must be eliminated to avoid fouling;
- The sensors must be removable, given that they will not last all along the full measurement period (at least until a solution is found);
- The front-ends must be close to the sensors (not farther than 10cm), but the electronics and the contacts must be watertight;
- The front-ends can be as distant as 90cm from the Smart Data Logger, but they can also be as close as 10cm, so the Smart Data Logger-Soil Probe connection wires should not bring analog or weak signals and the number of wires should be the smallest possible.

The Soil Probe design intends to consider all these constraints.

**Figure 3:**
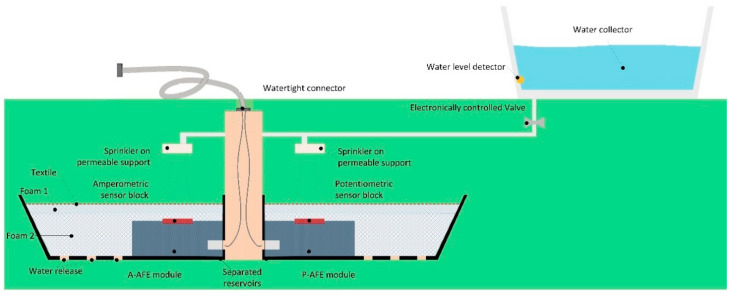
Soil Probe Architecture.

The Soil Probe (Figure 3) is composed by three groups of sensors, each one connected to an intelligent electronic front end (AFE).

Each front end contains the necessary analog circuits to adapt and filter the weak and noisy sensor signals. They also integrate a microcontroller to process and store the resulting values and to send these values to the Smart Data Logger on demand by means of a wired communication link that also provides the power supply.

There are two groups of sensors: a set of potentiometric sensors developed by one partner, and a set the amperometric sensors developed by another partner and there is one AFE per group or type of measurement. Each set of sensors is built on its own platform and connectivity configuration. The sensor blocks are detachable and disposable. The external side of the sensor block is permeable to the soil humidity, while the connector that links to the PCB meets strict requirements, such as allowing extremely low leakage current between electrodes being also fully watertight towards the electronics.

The soil reservoir, or cupola, is split in two parts, each part allocates an AFE; this should protect against (or minimize) potential leakage current between the blocks of sensors. Both sub-reservoirs are fixed to a central column that conducts the AFE wiring up to the external flexible cable that links to the SDL.

Each soil reservoir has holes in the bottom to allow releasing eventual water excess.

Covering each AFE module there are two layers of foam to attract the humidity around and help keeping wet the sensor region. A textile network prevents the reservoir from being filled with soil material.

The central column acts as support for the reservoirs and for the water sprinklers. Being open inside it also allocates the Smart Data Logger-Soil Probe cables.

A water collector, placed farther from the Soil Probe soil surface, might be useful to collect rain water. The release of water is dosed by an electronically controlled valve spraying along a configurable period of time before the measurement takes place. A liquid level detector is placed in the water collector to prevent from opening the valve if there is no water. A sprinkler is placed over each sensor module, so to throw sprayed water. The sprinkler surface would be open, for instance like a grid, so to allow passing through the water and fertilizers that will naturally arrive from rain and humanly programmed watering.

**Figure 4:**
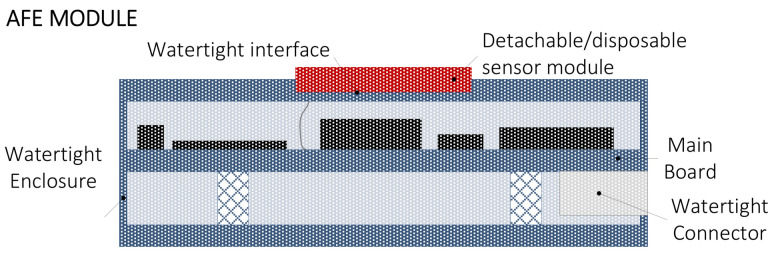
AFE module concept.

Figure 4 shows an overview of an AFE module.

The AFE module is watertight. The enclosure embeds a holder where to plug/unplug the detachable and disposable sensor block. The sensor block is connected internally to the AFE main board by means of a watertight interface.

The electronic board (or main board) is placed on an internal support screwed with columns. A watertight connector mounted on the PCB allows linking the AFE to the Smart Data Logger.

**Figure 5:**
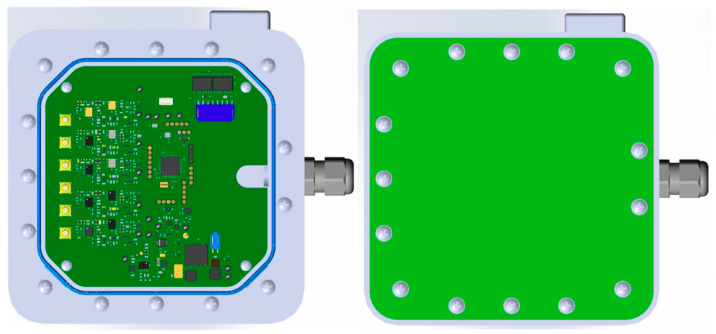
Potentiometric Sensor AFE mechanical file.

Figure 5 shows the final drawing of the Potentiometric Sensors AFE mechanical file.

The final module realized is quite big (160 × 160) mm^2^. This is due to the type of connector required to link the sensor electrodes and the electronic board, to the type of connector to link the Smart Data Logger, and to the enclosure style to provide robustness and ingress protection.

### 3.2. Air Probe

The integration of environmental data with soil information allows developing a more refined model that takes in account all the environmental factors that contribute to the crop and plant growth modelling. Moreover, data provided by the Air Probe are valuable for immediate advising of farmers about the optimization of resources according to climate conditions.

The Air Probe provides environmental data such as barometric pressure (STMicroelectronics Pressure sensor, LPS22HB (STMicroelectronics, Catania, Italy), commercially available), temperature and relative humidity (STMicroelectronics temperature and relative humidity sensor, HTS221 (STMicroelectronics, Catania, Italy), commercially available) and ultraviolet sensor, UVIS25 (STMicroelectronics, Catania, Italy), (a prototype, not commercially available) that are relevant to the plant growth and yield aspects.

In addition, the Air Probe provides information about soil gas emission by recognizing the gas type, its concentration and its emission rate. Nitrogen dioxide, methane and ammonia are mainly considered with the following target concentrations: 1–1000 ppm for CH4, 0–10 ppm for NH3, 0–100 ppm for N2O. STMicroelectronics Gas sensor, GHT25S (STMicroelectronics, Catania, Italy), (it is a prototype not commercially available), is introduced together with sophisticated algorithms for making it selective towards the targeted gasses. ST Gas sensor can be calibrated for single or more target gasses. For instance, if calibrated by ethanol in a range from synthetic air to 30 ppm, the accuracy is demonstrated to be 10% respect to a professional reference, with the embedded temperature sensor accuracy of ±0.5 °C, and the embedded relative humidity sensing channel accuracy of ±3.5%. Accuracy in the Air probe application versus the three target gasses is still under characterization.

This information can be used just to check that the soil fertilizers and nutrients have been absorbed in the soil, and to build up a refined growth plant model that can be used for growth prediction modelling and yield optimization as well.

Almost all the integrated sensors are commercially available. They have been selected because of their low power consumption, thus enabling easy spreading of IoT nodes within the field and increasing the amount of data collected from different areas.

**Figure 6:**
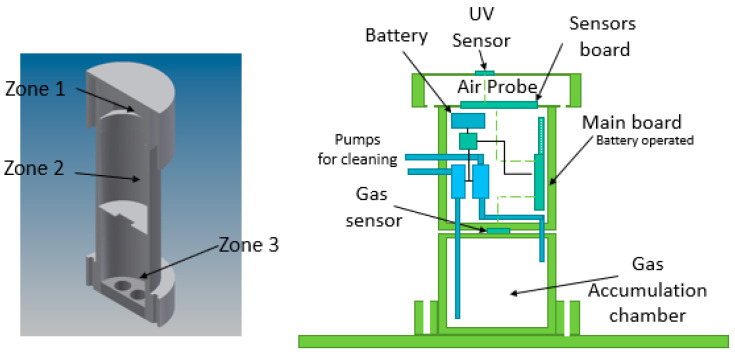
Air probe mechanical architecture.

The Air Probe has been designed taking in account all the guidelines and recommendations for environmental sensors with the aim to get out the right accuracy. Installation and use in a harsh environment subject to wind, rain, snow and farmer operations with different machineries has imposed extra design constraints and lead to more complexity. The mechanical architecture is represented in Figure 6.

As can be seen, three zones are defined: Zone 1 is the sensors zone with all environmental sensors (pressure, temperature, humidity and motion sensors) and the UV sensor close to the chamber edge for collecting IR radiation under a protective UV transparent protective layer; Zone 2 is the STM32L0 (STMicroelectronics, Catania, Italy), microcontroller-based control board with optional features such as battery and LORA connectivity, and with pumps module as well; and Zone 3 is the gas sensor zone that includes the gas sensor itself and the gas accumulation chamber. Zone 1 is designed for optimizing the air circulation, while Zone 3 is optimized for measuring gaseous emissions collected in the gas accumulation chamber. Both sensors chambers are separated and thermally insulated. The pumping systems doesn’t’ affect the pressure and temperature measurements that are executed in separated chambers. The function of the gas accumulation chamber is to accumulate the gas emitted from soil by increasing gas concentration on time. The function of pumps system is to clean up the gas chamber before measurement. One pump takes air from outside and puts it in the bottom part of the chamber, while the second one sucks air from internal side upper part to outside. They work simultaneously. The slope of the response curve after cleaning up the chamber is computed as soil emission rate, while the steady state level is computed as gas concentration. Gas sensors are individually calibrated for quantifying MOX (metal oxide) resistance values as ethanol or toluene equivalent concentrations. GHT25S (STMicroelectronics, Catania, Italy), is a combo device that contains temperature and humidity embedded channels for compensating MOX sensitivity changes to humidity and temperature variations as well by properly correcting calibration curves.

It is common to find design and installation where environmental sensors are affected by nearby electronics, heating sources and other external factors (e.g., pumps) that make inaccurate the sensor measurements. The design of the air probe sensor board has been optimized for insulating sensors from interferences and noise. In addition, Teflon is the material selected for gas accumulation chamber for avoiding influencing the gas concentration measurements by VOC released by the same accumulation chamber.

**Figure 7:**
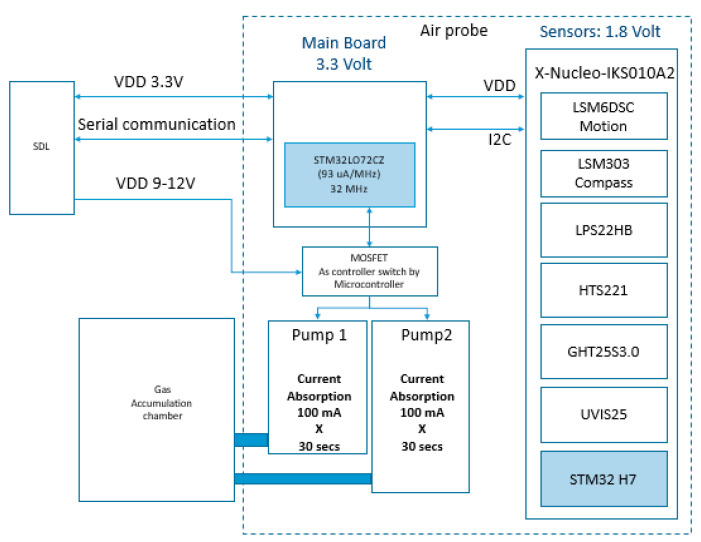
Air probe architecture.

Figure 7 reports the overall electronics architecture of the Air Probe.

All sensors are connected to the STM32L0 microcontroller by I2C digital communication interface. An additional STM32 works as co-processor for running artificial intelligent algorithms and other time-consuming processing routines. The SM32L0 drives the pump by controlling a MOSFET switch.

The Air Probe is featured with self-diagnosis. As already said, the Air Probe is designed for being used in harsh environment. It is important to keep the air probe in vertical position and in the right orientation. The gas sensor accumulation chamber collects gaseous emissions from the soil. The side space between the soil and the base crown must be close to zero, allowing to confine the emitted air into the chamber. On the other side of the probe, the UV sensor needs to be oriented in the appropriate direction. STMicroelectronics motion sensors such as LSM6DSL (STMicroelectronics, Catania, Italy), and compass SM303A (STMicroelectronics, Catania, Italy), are used for this purpose. Data coming from the motion sensors and from the compass allow to check the attitude of the Air Probe over time. In addition, the vibration of the pumps for cleaning the gas accumulation chamber is periodically verified by the low-cost accelerometer on board. This allows verifying that the pumps are working properly. The processing of attitude and pumps check is run on demand. The computation is done by the dedicated on-board microcontroller for running this kind of algorithms.

Finally, Metal Oxide Semiconductor (MOS) gas sensors are suitable for many applications in different industries. They can be considered low cost sensors in comparison with devices based on different sensing technologies that have higher pricing but even higher performances. Typically, the MOS gas sensors suffer of very low selectivity, and for this reason, are limited to applications where selectivity does not matter. To overcome this limitation, the Air Probe integrates a co-processor based on ST7 H7 microcontroller (STMicroelectronics, Catania, Italy), that runs all Air Probe processing routines, included some Artificial Intelligence algorithms for enhancing the gas sensor selectivity feature. This solution is based on the temperature modulation approach and data processing by a multi-layer perceptron neural network [24]. The details of this solution for Air Probe gas identification will be presented in Section 5.2.

Power consumption of the air probe depends on the specific sensor reading settings and overall strategy. A power consumption estimation is reported hereafter:

- Microcontrollers while running → 60 mA
- Reading sequentially of all data sensors except gas sensor → 1 mA per sensor in different time slots 3- Pump driving for cleaning up the accumulation chamber → 200 mA, 9 V for 30 s
- Reading gas sensor for gas quantification → 17 mA peak, 2 mA average
- Reading gas sensor for gas recognition → 17 mA in continuous mode for 60 s

### 3.3. Smart Data Logger

The Smart Data Logger (SDL) integrates all the features required to implement the application, namely, the acquisition and storage of the data generated by the Air Probe and the Soil Probe, the management of the rechargeable battery, its charger and a solar panel as harvester, a GPS as localizer, an accelerometer to estimate the attitude, and a simple user interface based on a button and three LEDs.

**Figure 8:**
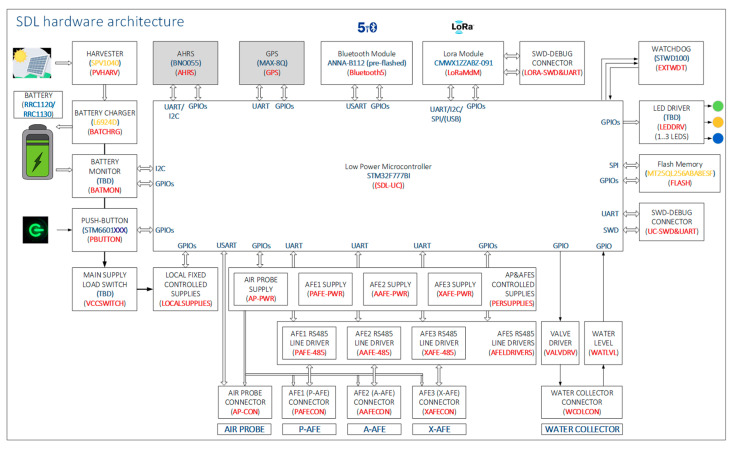
Smart Data Logger hardware block diagram.

Figure 8 shows the Smart Data Logger hardware block diagram.

The wireless connectivity is implemented using two wireless communication technologies: a Bluetooth5 module enabling the link to a Mobile Device, and a LoRa module with which the Smart Data Logger becomes an endpoint, connecting to the Back-End Server through a LoRa Gateway.

The solar cell is connected to a harvester that transfers the collected energy to a battery charger. The battery has been dimensioned with enough capacity to supply the node with the harvested energy along periods with no or not enough sunshine.

The selected type of battery pack embeds all the required safety protections and a fuel gauge based on Impedance Track^TM^ (RRC power solutions, Germany, Homburg) delivering accurate Time-To-Empty & Remaining Capacity predictions over the lifetime, readable with an I2C interface. The manufacturer provides all the compliance information.

A push button IC powered by the battery allows switching ON and OFF the node in a proper and controlled way. A physical push button managed by the user is the only control available in the node.

A load switch controlled by the push button IC enables or disables the main supply line. From this line on, several fixed supplies are generated as well as those that are controllable by the microcontroller. The microcontroller is able to switch OFF the node by ‘auto-killing’ via a simulation of the hardware push button.

The link with the Air Probe is composed by a controlled and protected power supply and a full duplex serial port with hardware flow control. A dedicated connector makes available the power supply and signals to the Air Probe linking cable.

There are two front-ends for the sensors, one for the potentiometric AFE and one for the amperometric AFE. They are located in the Soil Probe close to the sensors, the proximity being a strong requirement to minimize the potential noise. Given the particular requirements of each type of sensor, the AFE for the potentiometric sensors is developed separately from that for the amperometric sensors. The Smart Data Logger provides power supply and a UART to each one to communicate with it. In order to achieve isolation, each supply and communication port is linked to the AFEs by isolator ICs. In addition, the Soil Probe has been designed so to maximize isolation.

A third pair power supply and UART is available so as to allow adding a third AFE, making possible to add a set of commercial sensors to be able to compare performances and to add other required ones like a commercial moisture sensor.

There is one connector per AFE, all of different mode of insertion, to avoid erroneous connections. In any case the firmware is capable of recognizing the type of AFE connected and what its identity is. The same care is taken with other connected modules, sometimes by using other type of connector, sometimes by selecting connectors with different number of pins.

The Smart Data Logger integrates a valve driver and an input for the liquid level sensor, to implement the water collector. This module also has its own connector.

A Serial NOR Flash memory has been embedded to store the measurements, the device and connected modules status, all timestamped.

Three LEDs are available to provide basic status information on the module in stand-alone mode (with no link to a Smartphone or the Back-End Serve). Special care must be taken regarding their consumption, so better than being ON, they blink at a very low duty cycle.

A Real Time Clock (RTC) available in the selected microcontroller and supplied by the battery is implemented to allow timestamping with up-to-date date/time provided by the Mobile Device during configuration.

Two communication links are available and used according to the use case: a Bluetooth 5.0 module and a LoRa module.

For the Bluetooth 5.0 module, the u-blox ANNA_B112 (u-blox AG, Zurich, Switzerland) has been integrated, a quality module in small footprint and low power consumption.

For the LoRa module, the choice is mainly related to the great experience ST-I has in two modules where an STM32L microcontroller and Semtech radio are embedded: the Murata CMWX1ZZABZ-091 and the USI WM-SG-SM-42. The Murata module has been integrated.

Two additional but optional modules have been included: a GPS, to be able to provide the true node coordinates, and a 3-axis accelerometer, to be able to provide the device attitude.

An STM32F777BI microcontroller (STMicroelectronics, Catania, Italy) has been selected to be able to integrate all the described peripherals. This model integrates enough UART, SPI, I2C interfaces and GPIO to connect all of them.

**Figure 9:**
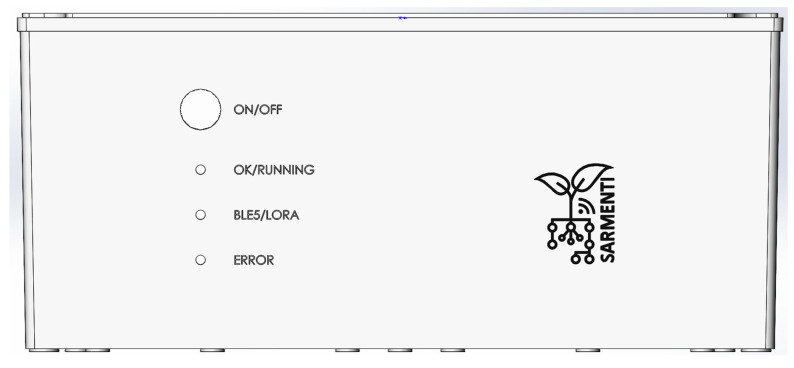
Smart Data Logger enclosure front view.

Figure 9 shows a front view of the Smart Data Logger, where the ON/OFF button and indicator LEDs are placed.

**Figure 10:**
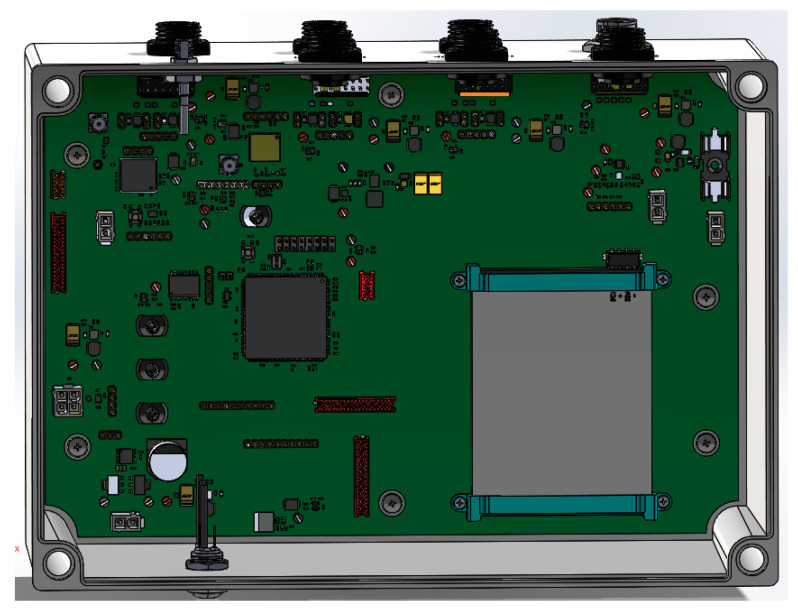
Smart Data Logger top view with the cover open.

Figure 10 a top view of the Smart Data Logger with the cover opened. It can be seen the electronic board fixed to the enclosure and some of the main components, the battery, the connectors, etc.

**Figure 11:**
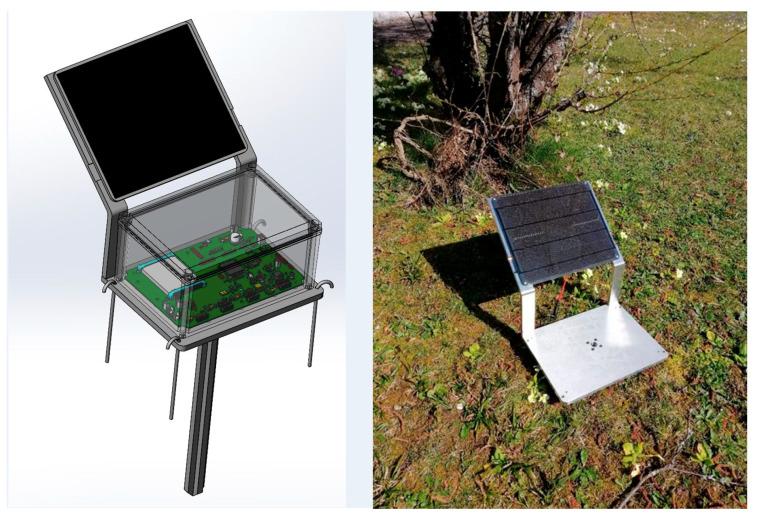
The Smart Data Logger and PV cell in the support, b-support fixed to the soil.

Figure 11 shows a schematic of the mechanical part manufactured to fix the Smart Data Logger and the Photovoltaic Cell to the soil.

**Figure 12:**
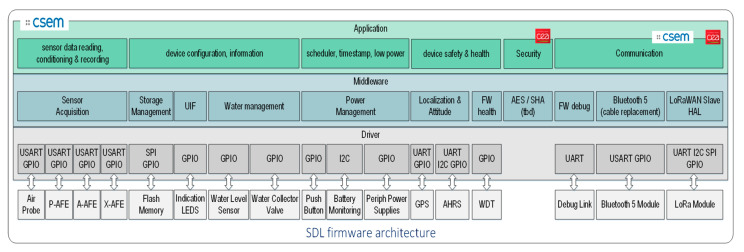
Smart Data Logger firmware architecture.

Figure 12 shows the Smart Data Logger firmware architecture.

It is based on a classical layered structure:

- The Driver layer is determined by the peripherals to be connected.
- The Middleware based on a scheduler, manages the general firmware functionality.
- The Application implements the use-case.

Protection against potential Cyber Attacks is integrated on top of the Application layer.

**Figure 13:**
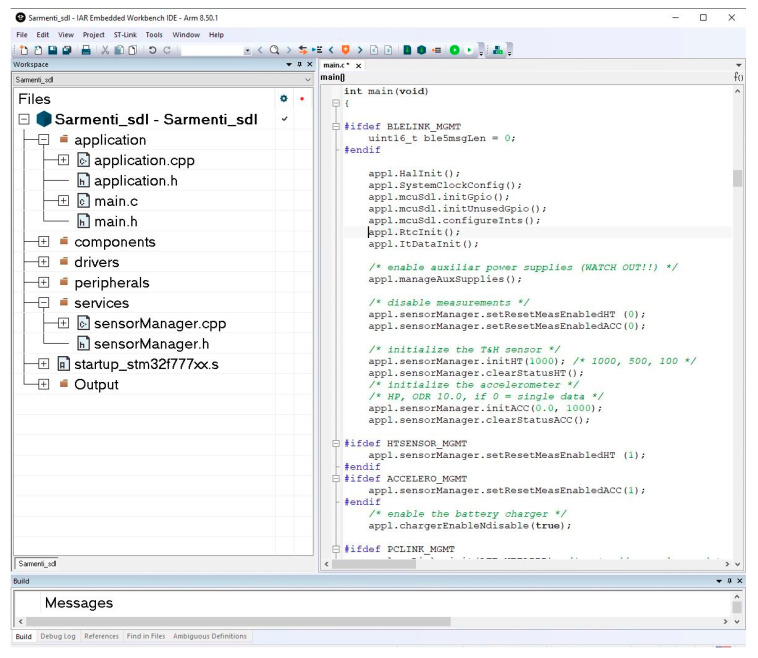
IAR Embedded Workbench IDE.

The firmware is developed using IAR Workbench (IAR Systems, Uppsala, Sweden). Figure 13 shows a view of the IDE and the Smart Data Logger layered firmware and part of the main routine.

A testing tool named PCTool was developed in C# to verify most of the Smart Data Logger features and to execute the acceptance tests before integration to the rest of the system. It also acts as node simulator for interfacing the Back-End-Server.

It should be highlighted that PCTool is capable of running functional, not performance tests, except for those performance tests related to timing and repeatability. In particular, the sensor performance testing is done by the sensor designers.

### 3.4. IoT Communication Path

**Figure 14:**
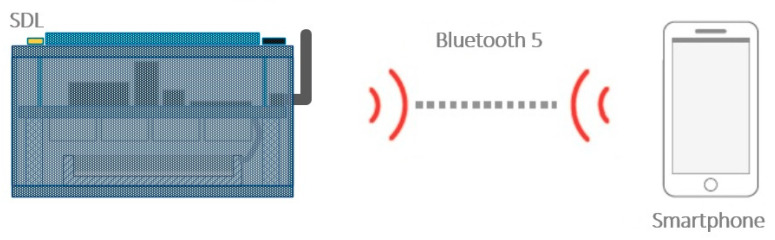
Smart Data Logger Bluetooth5 link to a Mobile Device.

The Smart Data Logger is basically an IoT (Internet of Things) node with two wireless communication means to meet different use cases. The Bluetooth5 communication (Figure 14) links the Smart Data Logger to a Mobile Device, i.e., a Smartphone running Android embedding a Mobile Application designed to configure the Smart Data Logger for the final use-case and to control it locally. The Mobile Application will be also used for testing purposes.

The profile offers two roles, where the Smart Data Logger is a GATT (Generic Attribute Profile) Server and the Mobile Device is a GATT Client. Some of the services implemented are the Device Information, the Current Time, Measurements, Settings, Autonomy and Storage Capacity.

**Figure 15:**
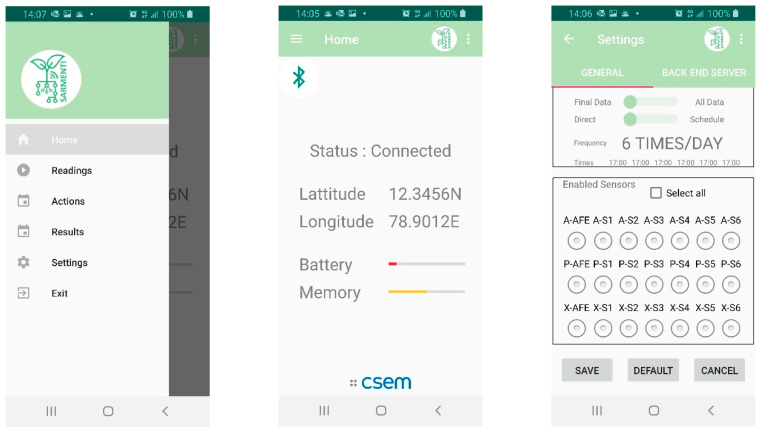
Some of the Mobile Application screens.

Figure 15 shows some examples of the Mobile Application screens, implementing the Smart Data Logger configuration, control and data reading.

**Figure 16:**
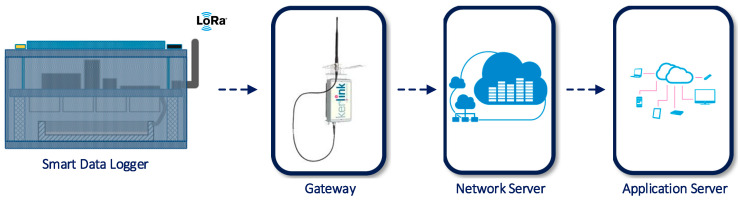
Smart Data Logger Network link to the Back-End Server using LoRa infrastructure.

The LoRa communication capability allows connecting all the Smart Data Loggers in the field to the Back-End Server by means of a LoRa Gateway with cellular link capability. The Back-End Server integrates the LoRa Network Server and the LoRa Application Server. Figure 16 synthesizes this link.

In this use-case, each Smart Data Logger sends the sensor data every day in a scheduled way to the Back-End Server. If the cellular link is not available at the scheduled time, it will retry to send it several times along the day.

## 4. Analytics and Decision Support

In SARMENTI, the analytics part proposes to inspect data produced and transmitted by the sensors (soil and air) and other connected devices (weather stations, air drones), together with previously available data sets and farmer know-how. The purpose is to discover and understand connections between data sets and then support the users (farmers) with decision support via predictions and prescriptions.

### 4.1. Data Analytics Approach

The analytics and decision support is based on a conceptual model that considers various inputs, such as a plant evolution model and environmental factors, historical data sets, etc. [25].

The plant evolution is a holistic topic that involves factors from multiple domains. Some studies state that the availability of nitrogen (N) and/or phosphorus (P) can influence the growth of plants in most ecosystems [8], therefore the initial conceptual plant model is focused on one of the dominant components, N. The efficient use of fertilizers could have an important impact both on the natural environment, but also on commercial gains. The decrease by 1% of N fertilizer use alone is estimated to lead to annual savings of $1.1 billion [26].

The prediction model is a multi-disciplinary problem that must consider the major impact factors upon the plant growth [27]. For conducting predictive analysis, the process model called Cross Industry Standard Process for Data Mining (CRISP-DM) [28] is used in the present work.

During the development of the initial plant models, several factors have been identified that directly influence plant growth. The initial plant models will consider these influencing factors iteratively, during the prototype development phase:

- Weather: probably, one of the most important environmental factors that impacts the crop lifecycle is the weather. It is preferable to have access to weather data as close to the farm location as possible, therefore the option of using on-premises weather stations is being analyzed;
- Soil Sensors Data: the availability of nutrients in the soil will have a huge impact on the plant growth model. The prediction model will consider these nutrients values, along with their own interconnections, e.g.,pH substantial influencer of nutrients available for plants.

The SARMENTI node will provide two redundant sets of soil sensors. Therefore, after the calibration and validation phase, the models can consider either to use both data points or to use the most stable sensor for specific ranges;

- Air Probe Data: the Air Probe can provide indication on the loss of specific nutrients via gaseous emissions, which is a loss of nutrients for the plants and can be very important factors of air pollution;
- Spectral Remote Sensing: the latest advances in agriculture development involve the use of unmanned aerial vehicles (UAVs) capable of multi-spectral or even hyperspectral remote sensing measurements for agriculture. It is important to consider this type of input in the decision/prediction model. If the farmers possess such technology, they can provide the results as input in the plant evolution model. These types of measurement could be used to measure variables such as soil condition, plant health and fertilizers;
- Farmer Feedback: the farmer will be able to provide input related to:
  ○ Plant reaching milestones;
  ○ Fertilizers: if fertilizers have been applied, if yes, which kind (natural, artificial), composition, quantity. Depending on these aspects, the fertilizer decomposition model should be used.

This input will allow the system to calibrate the model with the actual ground truth and also in time to calibrate the theoretical plant model for the specific farm conditions, thus achieving a better precision;

- Social feedback from similar farms: Cross-farm analytics will be included in the machine learning algorithm, especially for the similar farms: dimension, location, soil type, crops. This characteristic will be detailed in the next section.

### 4.2. Data Analytics Platform

The Back-End Server Platform is based on Atos Codex Datalake Engine [29] (Atos, Paris, France), which is a fully integrated Cloudera solution (Cloudera, Santa Clara, CA, USA). It is the first Datalake appliance fully virtualized with OpenStack (OpenStack Foundation, Austin, TX, USA), that is certified by Cloudera.

The appliance includes a comprehensive data management software stack, based on Cloudera Enterprise from Cloudera. The software stack represents a modern platform for machine learning and analytics optimized for the cloud and it leverages the Bull Sequana S200 server capabilities [30]. It provides the means to collect data from Internet-connected devices. It acts as a smart data hub, managing analytics and routing data streams to their specified applications for effective service use.

**Figure 17:**
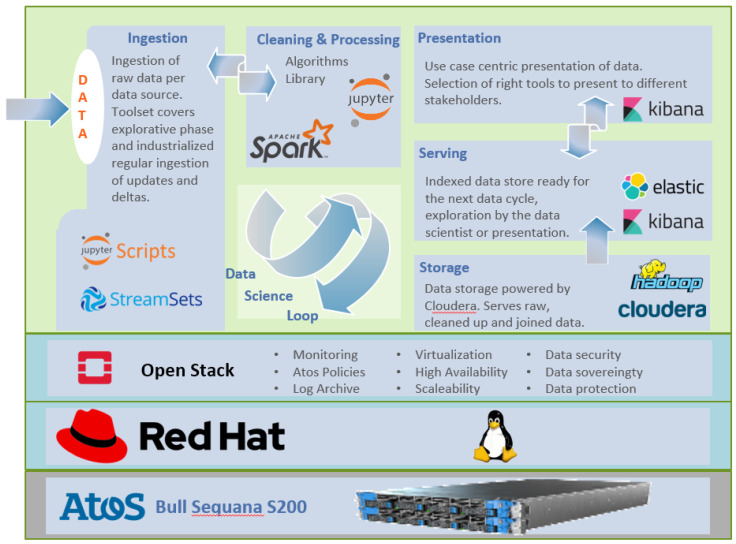
Data analytics software stack and the data science loop.

As depicted in Figure 17, the key software components are: Open Stack virtualization platform, RedHat enterprise Operating System, Cloudera Manager for Hadoop File System, StreamSets for flexible data ingestion and clean-up, Raw data storage in Hadoop File System with SW replication, Data clean-up, transformation via Jupyter during data science loop, Distributed data processing and analytics via Apache Spark2, Data serving and presentation with ElasticSearch and Kibana [31].

This figure also describes the data science loop which is based on a typical ETL process: extract, transform, load. This allows ingestion of raw data, cleaning and pre-processing before it’s available for visualization, as well for processing by the prediction and prescription algorithms. This data science loop allows iterating over the results of the analytics together with the expert users (agricultural project partners).

**Figure 18:**
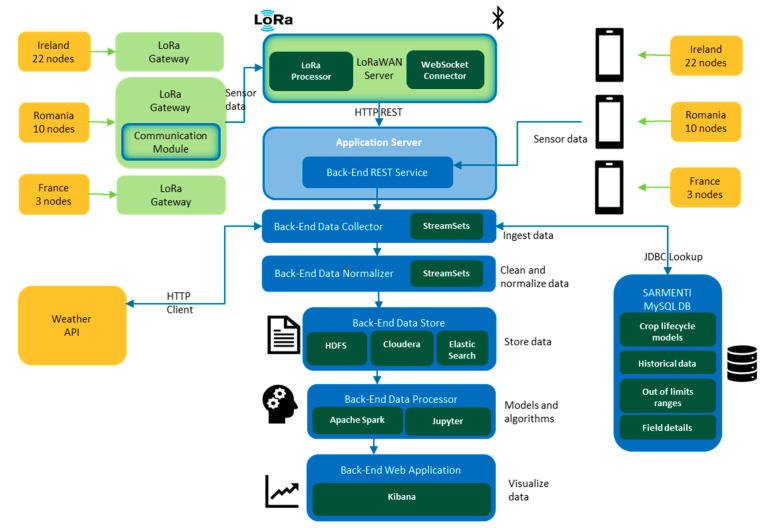
Analytics platform and communication paths from IoT devices.

Figure 18 further details the design of the analytics platform in the SARMENTI architecture. It also describes the communication paths and protocols between the IoT devices and the analytics platform.

### 4.3. Social Feedback Loop and Trust Index for Decision and Prediction Model

In a simple data analytics approach all data points are equal, which could be applied also for SARMENTI. After all, each node is virtually identical: the same number of sensors, the same output quality if calibration and drift corrections are successful, the same communication protocols. But in fact, the meaning of the data points they provide couldn’t be more diverse. For example, nodes with faulty sensors, different depth of the soil sensor, different field’s type or slope, influenced by external events, etc., could lead to false correlations or can wrongly influence the decision support. Coupled with the diversity of cultivated plants and their specific needs and characteristics, the complexity of the analytics increases exponentially.

This could be solved by simply having access to more data to cover each possible scenario with enough data points. Unfortunately, SARMENTI can only provide a few daily data points for each addressed plant type, but it has access to the know-how some excellent agri-partners which can provide valuable input.

Therefore, Trust Index [32] has been created for the data series, which, in its current initial version includes the relevance of the data point, the reliability of the sensors which have produced it and a social feedback.

**Figure 19:**
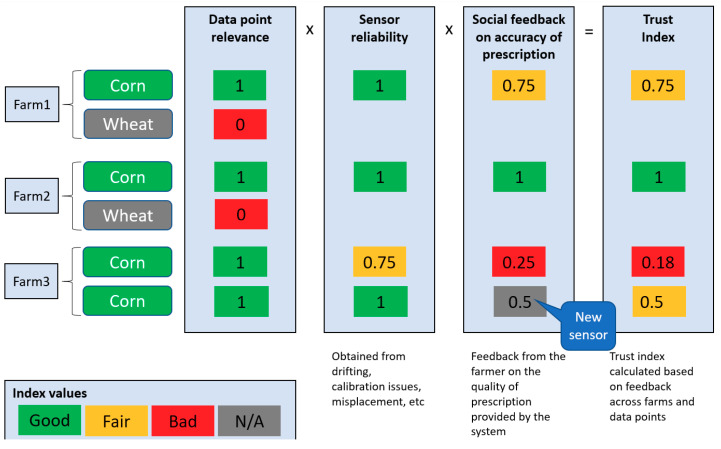
Social Trust Index–sample calculation for same crop type (Corn).

The Trust Index–currently in its initial stage–includes three main components, as illustrated in Figure 19 in a simplified form:

- Data point relevance
- Sensor reliability
- Social feedback

For example, for calculating a prediction, the algorithm considers as relevant a sensor value that is serving the same crop type, is placed in the same soil type or location-based similarities (neighboring farms). Furthermore, some values are identified as being doubtful because of drifting, calibration issues or in-field misplacement. These values receive a lower sensor reliably factor. Newly places sensors are by default considered in trial phase, so with a lower sensor reliability.

The platform is planned to use a social IoT approach in which similar farms can consider the previous crop cycle results, in which successful prescriptions have a larger weight than the ones invalidated by the farmers. The farmers can access the analytics platform via the web application called “SARMENTI Farm Advisor” that focuses on providing real-time predictions to the farmer. The application is optimized/adjusted for mobile devices with a small screen (phones, tablets) and offers the possibility to the user to:

- login with one of the roles defined (farmer, sensor manufacturer, administrator);
- visualize the recommendations and other data given by the prediction model;
- receive notifications about the suggested quantity of the fertilizer to be applied on the field;
- provide feedback for the prediction model in order to optimize it.

The analytics platform makes predictions and offers advices (prescriptions) to the farmer regarding various aspects, such as fertilizer consumption, crop growth rate, crop yield [33]. The final decision to follow the advices is on the farmer who can provide feedback about the prescriptions quality and the taken measures (e.g., quantity of applied fertilizers, plant evolution milestones, etc.).

The farmer social feedback on accuracy of prescription allows the analytics platform to learn from the agriculture expert farmers available in SARMENTI, as well as learning from a multitude of devices once the product is deployed in large numbers. This relationship between devices, their produced data sets, and the interaction with farmers makes the transition to a Social IoT.

**Figure 20:**
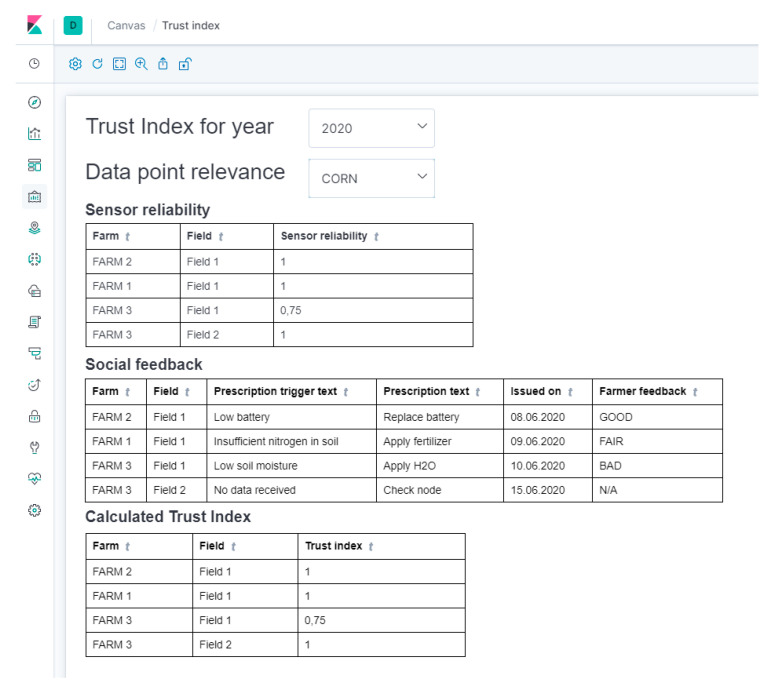
Initial Social Trust Index Visualized in the Analytics Platform.

This Trust Index can be visualized in the analytics platform, together with its components, as depicted in Figure 20.

**Figure 21:**
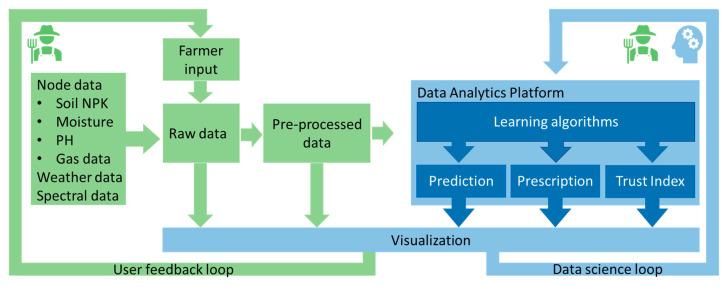
Analytics Platform: Data Flow, Main Processes and Feedback Loops.

Furthermore, the Social Trust Index is at the confluence between the two feedback loops of the system: the user feedback loop in which the farmer provides feedback, and the data science loop in which the data analyst and the farmer analyze the learning algorithms and make adjustments and improvements. The Trust Index components and values are described in a simple JSON file, making adjustments easy to do, as simple as editing a text file, as seen in the except below:

“SocialFeedback”: [
{“Name”: “Good”, “Weight”: 1},
{“Name”: “Fair”, “Weight”: 0.75},
{“Name”: “N/A”, “Weight”: 0.5},
{“Name”: “Bad”, “Weight”: 0.25}],

The Trust Index allows the creation of a virtuous circle in which every good prescription is reinforced, as depicted in Figure 21.

## 5. System Integration and Preliminary Results

### 5.1. System Integration, Verification and Validation

Verification of each system component is performed under laboratory conditions, including the nutrient sensor calibration with agronomic soil tests and establishing gas sensor range of concentrations.

**Figure 22:**
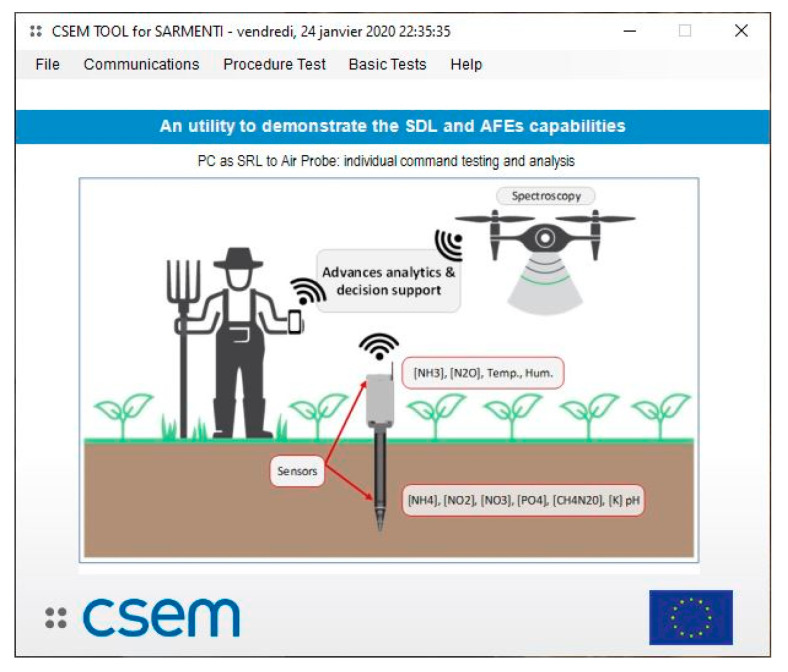
Smart Data Logger PCTool main screen.

Figure 22 shows PCTool main screen, use to perform functional testing. PCTool development was started by CSEM in the frame of previous EU projects as testing tool and features have been added for SARMENTI.

PCTool is capable of directly communicate with each one of the sensor electronics, with the Air Probe, and with the Smart Data Logger, in order to verify each communication protocol and most of the functionalities.

**Figure 23:**
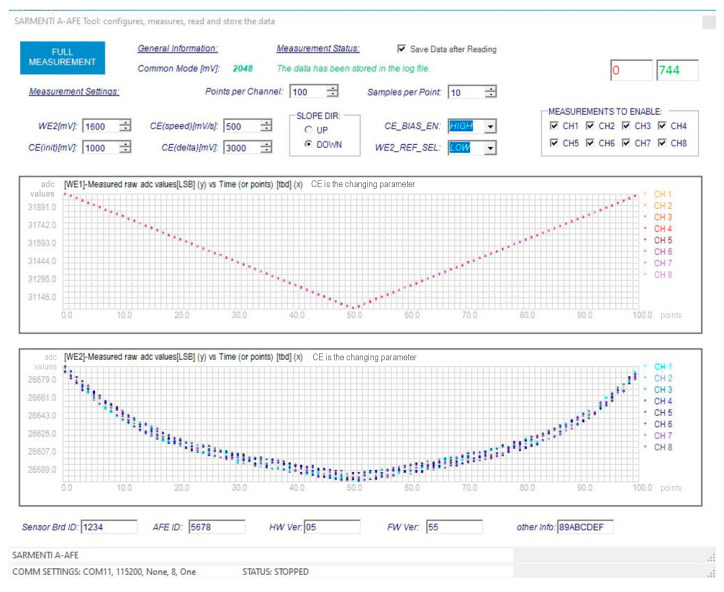
Measurement values in the SDL PCTool.

As an example, Figure 23 shows a screen to test one of the Soil Probe group of sensors. It allows configuring the internal circuit and to launch a test to measure the sensor values and to read the results. The test is configurable, allowing the measurement and reading of one, several or all the channels. The measurements are done at two places of the circuit, one hundred points are measured per channel and per place. Each chart shows the results of one place for all the enabled channels.

Concerning the PCTool verification, for instance for the case illustrated in Figure 23, the values sent by the amperometric AFE represent the voltage at the ADC input (measurable using a calibrated multi-meter), represented in the chart Y-axis for all the channels and for each value of CE (a variable voltage which is also measurable). The read values are stored in csv files, where the values measured with the multi-meter are also entered and compared at least a first time. The verification is done a defined number of times and for different boards, so it is possible to gain confidence in the whole chain: that the AFE sends correctly the values, that the PCTool understands, shows and store correctly the values. PCTool does not verify that the sensor measures always the same value for the same nutrient concentration nor that the analog part of the front end reads correctly the sensor. The first verification is done by the sensor designer and the second is done by the AFE designer in lab.

Following individual functional and performance tests, the system is end-to-end validated (sensing elements, Smart Data Logger, cyber-security implementation, data processing and decision support (at edge and Back-End-Server levels). The integration strategy is guided by the critical path of data communication, to deliver sensor values from the Air and/or Soil Probes to the user. Therefore, components are incrementally integrated one by one, until this critical path is built. Verification activities are performed along the bottom-up integration activities.

The system validation is done under growing conditions with a crop (growth chamber) and its ability to capture temporal concentrations under environmental stresses of drying-rewetting cycles, temperature fluctuations, etc. Evaluation of the system robustness and reliability at sensing soil nutrient concentrations and gaseous emission will be carried out across a range of field sites (Ireland, Romania and France), representative of pedoclimatic conditions and crop types in Europe.

### 5.2. Preliminary Results

Project preliminary results are obtained at all levels of the solution: sensors, integration part (presented in Section 3.3 including embedded system design and firmware architecture) and data analytics.

One of the results of the SARMENTI system is related to the Air Probe that have the capability to measure gas concentration and to identify the gas [34] type based on GHT25S sensor and data processing by AI algorithms.

**Figure 24:**
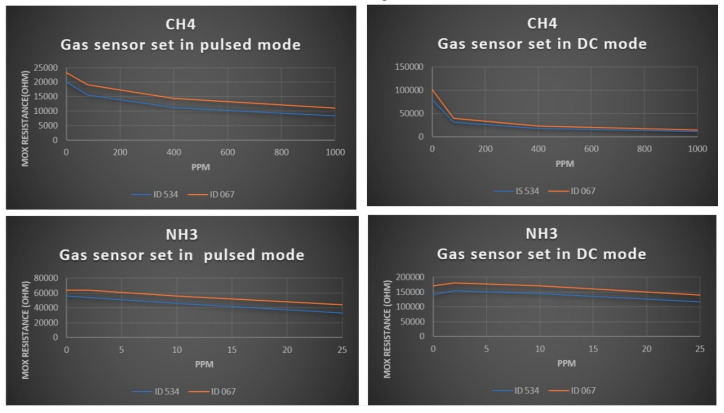
Gas sensor in the air probe showing direct relation between MOX (metal oxide) resistance and gas concentration.

First, we report here some results related to the gas sensor tested in a gas chamber with continuous flow of Methane and Ammonia at different concentrations. The gas sensor has been set in two working modes, namely, high-accuracy continuous mode (DC) and lower-accuracy by pulsed mode (PM), this latter with one reading per second. Figure 24 reports in the x-axis the concentration and in the y-axis the MOX resistance value in ohm for two gas sensors identified as ID534 and ID067 in DC and PM modes. As expected, the MOX resistance clearly decreases when the gas concentration increases. These curves are even used as basis for single sensor calibration.

**Figure 25:**
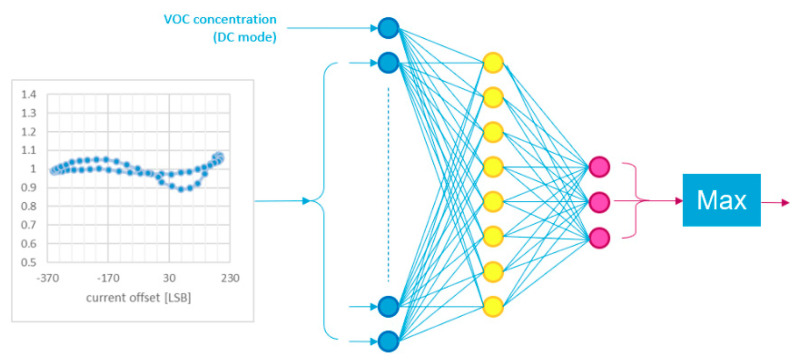
MLP neural network for gas identification.

The Air Probe embeds a co-processor based on STM32 H7 microcontroller that runs all the Air Probe processing routines by including the Artificial Intelligent algorithms for enhancing the gas sensor selectivity feature. MOX sensors are typically sensitive to many gasses but it is not possible to distinguish one target gas from others. The air probe approach is based on the MOX sensing material temperature modulation and data processing by a multi-layer perceptron (MLP) neural network. The neural network is formed by an input layer with same number of extracted features and output layer with a number of neurons equal to the target gasses to be identified. The number of neurons in the hidden layer is determined by iterations with the aim to minimize the classification error. Extracted pattern called gas fingerprint is formed by a single computed MOX resistance value in DC mode and multiple time discrete values of MOX resistances coming from micro hot plate temperature modulation. Temperature modulation is executed by sensor internal current level driving circuitry. The MLP neural network is depicted in Figure 25. MOX resistance values filtered from DC component and its 1st harmonic are reported in y-axis, while the driving current level in least significant bit (LSB) is in x-axis. This combination represents the input dataset for the MLP neural network.

The maximum value from all output neurons (last block in Figure 25) identifies the related gas type. As example, in the next reported case, we have developed a specific multi-layer perceptron neural network for the Air probe with 51 neurons as input layer, 8 neurons as hidden layer and 8 neurons as output layer. Extracted features as input are the single computed gas concentration MOX resistance value in DC mode and 50 values of MOX resistances coming from micro hot plate temperature modulation by internal current level driving circuitry.

**Figure 26:**
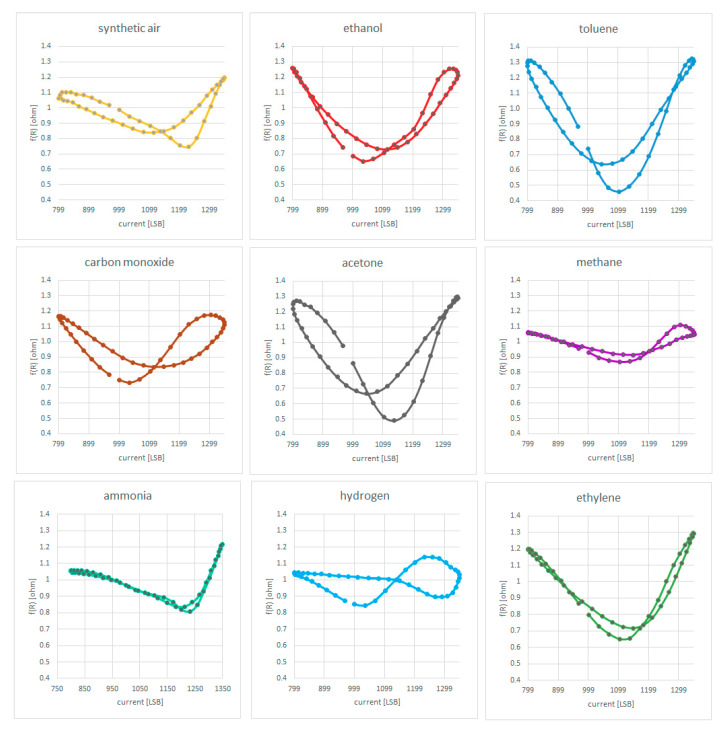
Gas fingerprint used for AI gas identification.

MOX resistance values from DC component and its 1st harmonic represent the input dataset for the MLP. In each graph in Figure 26, x-axis shows the current level in LSB, while y axis represents the MOX resistance value filtered from DC component and its 1st harmonic called f(R). The MLP has been trained with the dataset reported in Table 1 with the aim to recognize these gas fingerprints on field.

Preliminary results are really promising since the classification error has been less than 5% for all gasses.

Some other attempts of selectivity enhancement are reported in [13,14,15,35].

On the Back-End-Server data analytics side, data mining algorithms were produced for the plant model that generated the consolidation of a prediction model for main nutrients like nitrogen.

**Figure 27:**
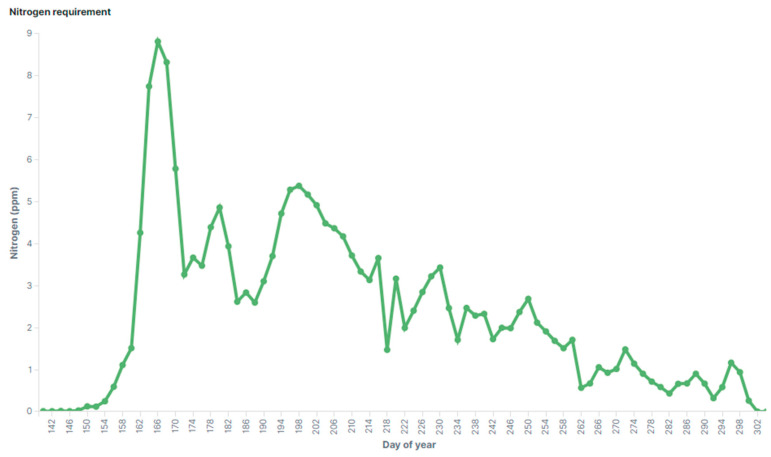
Conceptual Model—Plant’s daily nitrogen need.

Based on available agricultural data, several initial conceptual plant models have been created. One of them reflects the plant needs in terms of nitrogen. A visualization of such model for corn can be seen in Figure 27. Similar models describe other plant characteristics, for example leaf area, number of leaves, etc.

**Figure 28:**
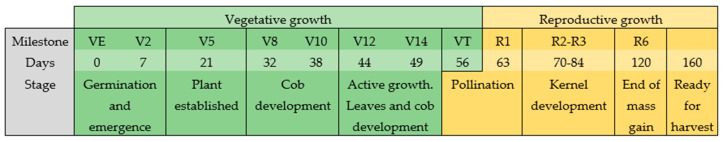
Maize growth stages [36].

Additionally, seed providers offer information regarding plant growth patterns, including ways to visually identify some “milestones”. As an example, Figure 28 shows these milestones and their timings for corn.

By using typical classification algorithms such as linear regression or support vector machine patterns already emerge regarding plant nutrient needs. For example, early plant growth requires high levels of soil nitrogen. Similar patterns were observed in relation with the plant leaf area in the initial stages of plant growth.

**Figure 29:**
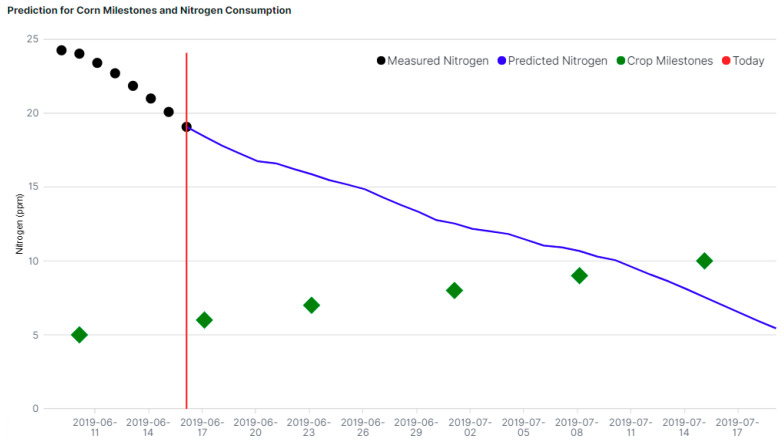
Simulation of prediction for corn milestones and nitrogen consumption.

The plant model, together with special plant growing characteristics (growth milestones, soil pH, farmer feedback, weather, etc.) are analyzed in the data analytics module and a prediction is generated for the plant evolution and projected nutrient consumption, which can be observed in the Figure 29 for nitrogen.

Based on the prediction for the milestones and nutrient consumption, prescriptions are generated for the farmer to perform corresponding in-field actions. These prescriptions are transmitted via a notification mechanism. The learning algorithm is re-enforced via feedback from the farmer who can validate/invalidate the prescription based on agricultural best practices and his experience. This is performed via the web application called “SARMENTI Farm Advisor” described in Section 4.3.

So far, all observations have been made on data sets available from previous experiments done by SARMENTI agri-partners. Of course, further data analytics work is planned to be done with data sets produced by the SARMENTI sensors during testing and validation stages, both from growth chambers and field growth.

## 6. Conclusions

As in most industries, digital technologies represent a lever for innovation also in the agriculture industry. Parameter monitoring doubled by data analytics that can lead to recommendations for farming actions, may increase productivity and even protect the environment with the help of gaseous emissions monitoring. The most efficient way to evaluate the in situ parameters is via sensing with dedicated elements.

This paper is presenting a multi-sensor IoT node that intends to measure soil nutrients and gaseous emissions just above the soil, the integration methods and communication path towards an analytics platform that provides advices to the farmer about the fertilization strategy. The paper is presenting the multi-sensor node architecture at hardware and software level, consisting on three main elements: a Soil Probe, a Gas Probe and a Smart Data Logger. The section describing the node components presents also relevant physical and mechanical sensor design, that has to take in consideration the challenging set of constraints of in-field deployment including power and communication limitations. Details of the custom integration module, part of the Smart Data Logger, are presented, including embedded system design and the firmware architecture.

The paper results related to the multi-sensor node are focused on the presentation of the gaseous sensing element, that measures gas concentration and is able to identify the gas type based on GHT25S sensor and data processing by AI algorithms. We have detailed the multi-layer perceptron (MLP) neural network used for the innovative gas fingerprinting algorithm with gas classification errors of less than 5%.

We have also emphasized the methods for testing and validation during different phases of the project, so we have considered relevant to present the developed test tool named PCTool that emulates most of the data running through the Smart Data Logger.

Furthermore, we have detailed the architecture and functionality of the Back-End Server in charge of data storage and analytics, a typical computing element where relevant crop information can be modeled using machine learning algorithms. The paper illustrates the way the conceptual plant model was modeled in software including the milestones in the evolution of the crop (exemplified for corn). The paper presented an integrative graphical representation of the prediction for nitrogen consumption in relation to the corn evolution milestones. These models are decision triggers, as the Back-End Server has also the role of providing real-time visualization and prescriptions via the “SARMENTI Farm Advisor” web application and the mobile implementation presented in the paper. We have emphasized the importance of the farmer feedback to our “Farm Advisor” decisions that is a relevant input/evaluation of the reliability and correctness of our automated recommendations. In order to assess the actual reliability of the sensing values, a Social Trust Index is used at the confluence between the two feedback loops of the system: the user feedback loop in which the farmer provides feedback, and the data science loop in which the data analyst and the farmer analyze the learning algorithms and make adjustments and improvements.

To the best of our knowledge, there are no other complete systems that provide an end-to-end eAgriculture implementation, from real-time data acquisition based on sensors up to prediction and prescription of farming actions based on a complex series of parameters that include the biological model of the crop and environmental considerations. As part of our future work, we envision the extension of the decision and prediction algorithm with collaborative feedback, once we extend the experimental area to multiple farms and leverage on farm similarities based on, for example, location, crop type or soil type. At this point, the project is focused on laboratory tests, growth chambers experiments and limited paddocks tests. In the future, the work will include more social interaction and reputation-based classification of decisions.

## Figures and Tables

**Table 1:** Training set.

Target Gas	Gas Concentrations [ppm]	Dataset Number
air		12
ethanol	30, 10, 2	9
methane	400, 2000, 100	9
toluene	25, 10, 2	9
carbon monoxide	250, 100, 20	9
ethylene	50, 20, 4	9
acetone	5, 2, 0.4	9
ammonia	50, 90, 10	9
hydrogen	1000~5000	6
